# Laparoscopic Surgery for Intrahepatic Cholangiocarcinoma: A Focus on Oncological Outcomes

**DOI:** 10.3390/jcm10132828

**Published:** 2021-06-26

**Authors:** Francesca Ratti, Andrea Casadei-Gardini, Federica Cipriani, Guido Fiorentini, Federica Pedica, Valentina Burgio, Stefano Cascinu, Luca Aldrighetti

**Affiliations:** 1 Hepatobiliary Surgery Division, IRCCS San Raffaele Hospital, 20132 Milano, Italy; cipriani.federica@hsr.it (F.C.); fiorentini.guido@hsr.it (G.F.); aldrighetti.luca@hsr.it (L.A.); 2 Department of Medical Oncology, IRCCS San Raffaele Hospital, 20132 Milano, Italy; casadeigardini@gmail.com (A.C.-G.); burgio.valentina@hsr.it (V.B.); cascinu.stefano@hsr.it (S.C.); 3 Department of Experimental Oncology, Pathology Unit, San Raffaele Hospital, 20132 Milano, Italy; pedica.federica@hsr.it

**Keywords:** laparoscopy, minimally invasive, intrahepatic cholangiocarcinoma, new technique, liver resection

## Abstract

Background: The aim of the present study was to analyze the long-term outcomes of laparoscopic and open surgery for intrahepatic cholangiocarcinoma (iCCA) in a series, collected in a tertiary referral center with a high annual volume of laparoscopic activity. Methods: Between January 2004 and June 2020, 446 liver resections (LR) were performed for iCCA: of these, 179 were performed by laparoscopic surgery (LS) and 267 with the open approach. The two groups were matched through a 1:1 propensity score using covariates representative of patient and disease characteristics. The study and control groups were compared, with specific attention given to oncological outcomes (rate of R0, depth of resection margins, overall and disease-free survival, rate, and site of recurrence). Results: The number of retrieved nodes, rate, and depth of negative resection margins were comparable between the two groups. The interval time between surgery and subsequent adjuvant treatments was significantly shorter in LS patients. No differences were shown even in the comparison between the LS and the open group in terms of median disease-free and overall survival. Moreover, the disease recurrence rate was comparable between the LS and the open groups (45.2% versus 56.7%), and the recurrence pattern was similar. Conclusions: The minimally invasive approach for iCCA was once again confirmed to be associated with advantages in terms of intraoperative and short-term outcomes, but was also proven to be oncologically non-inferior to the open counterpart. In the present study, overall and disease-free survival were found to be similar between the two approaches.

## 1. Introduction

Surgery is the only potentially curative treatment for intrahepatic cholangiocarcinoma (iCCA); the prerequisites—to be effective and associated with a long-term advantage for patients compared to non-curative treatments—include the radical excision of the disease with negative margins (frequently requiring major hepatectomies) and the removal of the locoregional lymph nodes of stations 8 and 12 [1,2,3,4,5]. These features are among the reasons why cholangiocarcinoma was later analyzed and described in the context of minimally invasive surgery [6,7,8] compared to other malignant tumors of the liver, such as hepatocellular carcinoma [9,10] and colorectal liver metastases [11,12]. Indeed, indications of the role of laparoscopy in this area were not provided in the Southampton European consensus guidelines [13], while the literature has been enriched only in recent years with progressively larger series and studies comparing minimally invasive approaches with the open technique for iCCA [14,15]. Recently, therefore, reduced blood loss and blood transfusions, reduced postoperative morbidity, and faster postoperative recovery compared to the open technique are some of the advantages that have also been confirmed in the laparoscopic surgery of iCCA [6,7,8,14,15].

Furthermore, the keypoints of iCCA surgery—initially deemed to be the most critical issues for initiating the large-scale diffusion of this technique—were specifically targeted to exclude their role as a real limit to the implementation of the laparoscopic technique. In particular, hot topics that deserved dedicated insight were the technical feasibility of major hepatectomies for large ICC nodules [16] and the adequacy of laparoscopic lymphadenectomy compared to the traditional open technique [7]. Indeed, the indisputable short-term advantages conferred by minimal invasiveness can avoid clashing with the achievement of adequate radicality, therefore jeopardizing the oncological efficacy of surgical treatment.

While for hepatocellular carcinoma and colorectal metastases the non-inferiority of the laparoscopic technique compared to the open one in terms of long-term outcomes is now a consolidated finding [9,10,11,12,13], the relative delay in the spread of laparoscopy for iCCA has resulted in a paucity of data regarding the comparative long-term results of using the open and laparoscopic approaches for this disease.

The primary endpoint of this study is therefore to evaluate, in a comparison between groups after a 1:1 propensity score matching, the long-term outcomes of laparoscopic and open surgery for iCCA in a series collected in a tertiary referral center with a high annual volume of laparoscopic activity. Our secondary purpose is to evaluate any differences conferred by the technical approach in intraoperative and short-term postoperative outcomes.

## 2. Methods

### 2.1. Study Design

The clinical, pathological, and short-term and long-term outcome data of patients undergoing surgical treatment for iCCA at the Hepatobiliary Surgery Division, San Raffaele Hospital, Milan, were prospectively collected and then retrospectively analyzed for the purposes of this study.

**Figure 1:**
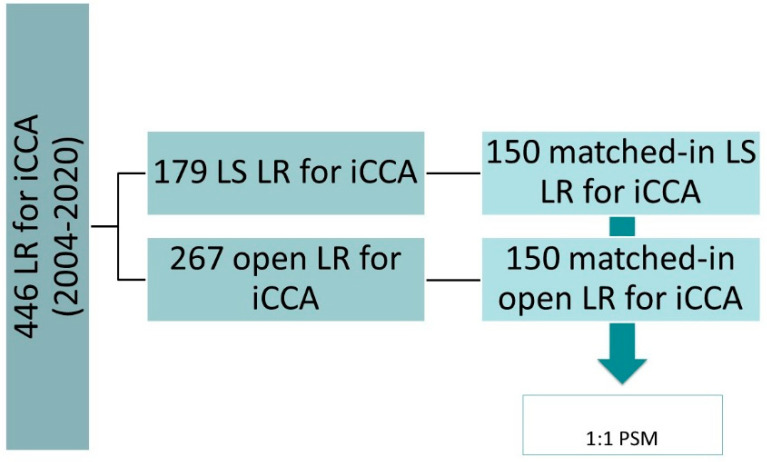
Study design. Abbreviations: iCCA, intrahepatic cholangiocarcinoma; LS, laparoscopic surgery; LR, liver resection; PSM, propensity score matching.

Patients with the following characteristics were excluded from the analysis: patients with Klatskin tumors or periductal infiltrating and intraductal cholangiocarcinomas, gallbladder cancers, unconfirmed iCCA on final pathology report, and less than 6 months of follow up. Between January 2004 and June 2020, 446 liver resections (LR) were performed for iCCA, of which 179 were performed using laparoscopic surgery (LS) and 267 with the open approach. The two groups were matched through a 1:1 propensity score using covariates representative of patient and disease characteristics. In this way, the study group (LS Group, *n* = 150) and the control group (open group, *n* = 150) were obtained. To fulfil the main endpoint, the study and control groups were compared, with specific attention given to oncological outcomes (rate of R0, depth of resection margins, overall and disease-free survival, rate and site of recurrence). The study design is shown in Figure 1. The standard implementation of the laparoscopic approach for iCCA was performed in our center between 2014 and 2015. Following this period, patients were standardly recruited to the laparoscopic approach unless presenting with one of the following characteristics: lesions requiring biliary or vascular resections; patients with lesions infiltrating the inferior vena cava; patients with lesions in contact with the hepatic vein of the future liver remnant (if only one vein was remaining in the remnant liver).

### 2.2. Treatment Strategy and Surgical Technique

Before surgery, all patients were evaluated by thoracoabdominal imaging (computed tomography and magnetic resonance imaging) and blood tests, including serum concentrations of tumor markers (carcinoembryonic antigen, Ca 19.9). Selected patients also underwent positron emission tomography (PET) imaging to rule out the presence of extrahepatic disease. Treatment strategies were routinely evaluated at weekly multidisciplinary meetings, during which liver surgeons, radiologists, hepatologists, and medical oncologists defined the indications for surgical procedures as well as the type and technique of resection [17].

Open approach—Under general anesthesia, xipho-supraumbilical incision extending to the right flank was performed in patients undergoing open LR.

Laparoscopic approach—Under general anesthesia, with the patient’s legs apart and the first surgeon standing between them with one assistant at each side, a five-ports standard configuration (inverted J shape) was used.

In both the open and LS approaches, intraoperative ultrasound was performed routinely to assess the liver anatomy and to confirm resectability and the relationship between the lesion and major liver structures. Liver resection was performed by alternating the use of the ultrasonic dissector (Sonosurg, Olympus, Tokyo, Japan) and bipolar forceps, exposing the vascular structures, then coagulating or selectively sealing with clips or staplers, depending on the size. A formal lymphadenectomy was performed involving the complete removal of lymph nodes of stations 8 (on the common hepatic artery) and 12 (including regional nodes 12a along the hepatic artery, 12b along the bile duct, and 12p behind the portal vein) [7]. The Pringle maneuver was used as needed to control intraoperative bleeding.

Histological staging was performed according to the TNM classification following the criteria of the Seventh Edition of the American Joint Committee on Cancer (AJCC) [18]. R0 resection was defined as the absence of tumor cells on resection margins.

### 2.3. Data Collection and Outcome Evaluation

Data regarding the patient’s preoperative and disease characteristics were collected, as well as intraoperative and pathological findings. Satellites are defined are lesions in close proximity to the primary tumor mass, while multiple lesions are those showing tumors arising in distant segments. The oncological risk of short-term recurrence was calculated for all the patients, using the postoperative prediction model of very early recurrence (VER) after hepatectomy for ICC (available at https://k-sahara.shinyapps.io/Veryearly-recurrence/ (accessed on 10 April 2021).

The overall complication rate at 90 days was reviewed and evaluated and complications were classified according to the Dindo–Clavien classification [19]. Mortality was defined as any death during the postoperative hospital stay or within 90 days of resection. The length of hospital stay and the time to full functional recovery were recorded and analyzed. Functional recovery was defined as the achievement of the following: adequate oral feeding, adequate pain control with oral analgesics, normal deambulation and self-care autonomy, and no complications. Data regarding follow-up, survival status, and occurrence and type of relapse were analyzed. Three- and five-year overall survival (OS) and disease-free survival (DFS) were assessed using the Kaplan–Meier method.

### 2.4. Statistical Methods

To minimize the effect of bias and ensure the highest possible level of evidence in the context of a retrospective study, a 1:1 propensity score matching with a small caliper (0.2) was performed, taking into consideration all covariates which might have affected the selection and indication to the LS or open approach or the oncological outcomes. Those covariates included age, ASA (American Society of Anesthesiology) score, chronic liver disease (presence/absence), Ca 19.9 serum level, tumor dimension at preoperative imaging, number of lesions, and tumor stage. After matching, all variables were compared using the *χ*^2^ or Fisher’s exact test for categorical data, the Mann–Whitney U test for non-normally distributed continuous data, and the Student’s *t*-test for normally distributed continuous variables. All data were expressed as the mean plus or minus the standard deviation or median and range. Survival curves were generated and compared using the Kaplan−Meier method. Significance was defined as *p* < 0.05. All analyses were performed using the statistical package SPSS 26.0 (SPSS, Chicago, IL, USA).

## 3. Results

### 3.1. Descriptive Data

Patients and disease characteristics after propensity score matching are reported in Table 1. Age, sex, biometrics, and comorbidities were similar between the LS and the open groups. Underlying liver impairment or cirrhosis secondary to viral infection and/or alcohol-related damage was present in 30% of patients in the LS and 24% in the open group (*p* = ns). All patients with liver impairment or cirrhosis had preserved liver function (Child A). Forty-four patients in the LS group had previous abdominal surgery (either open or laparoscopic) in their medical history. Most patients in both groups (72% in the MILS and 70% in the open group) had a single liver lesion. Lesion diameter was similar in the LS with the open series (respectively 5.3 ± 2.3 cm versus 5.8 ± 1.2 cm, *p* = ns). Eighty-eight percent of patients in the LS and 90% in the open group had intraoperative nodal dissection. Final pathological examination demonstrated nodal metastases in 56 out of 132 (42.4%) patients in the MILS group and 53 of the 135 (39.2%) patients who had undergone lymphadenectomy in the open group. Chemotherapy with neoadjuvant intent was administrated in a comparable proportion of patients in the laparoscopic compared with the open series (3.3% versus 4.7%). The postoperative VER risk was low, intermediate, and high in 10.6%, 56.7%, and 32.7% of patients in the LS group and 9.3%, 55.3%, and 35.3% in the open group.

### 3.2. Short-Term Outcome Data

Operative characteristics are shown in Table 2. Major hepatectomies (removal of ≥3 liver segments) were performed in 51 (34%) (in detail: 24 right, 25 left, and 2 central hepatectomies) and 55 (36.7%) (in detail: 26 right, 27 left and 2 central hepatectomies) patients in the MILS and open groups, respectively (*p* = ns).

The length of surgery was comparable between the two groups. Laparoscopic procedures were successfully completed in 133 patients, whereas the procedure was converted to laparotomy in 17 patients. Concerns regarding oncologic inadequacy (10 cases), bleeding (6 cases), and adhesions (1 case) were the causes of conversion. Significantly reduced intraoperative blood loss was recorded in the LS (mean 150 ± 100 mL) compared with the open group (mean 350 ± 250 mL) with *p* = 0.021, despite the comparable intraoperative use of the Pringle maneuver between the two groups (88.7% in the LS and 90.7% in the open group).

The median number of retrieved nodes, the achievement of negative resection margins, and the depth of surgical margins on liver parenchyma were comparable between both groups.

Postoperative overall complications rate and mortality are reported in Table 2. Postoperative complications occurred in 14.7% of patients in the LS group and in 24% of patients in the open group (*p* = 0.015). In particular, the incidence of minor (Dindo–Clavien < 3) complications was significantly lower in the LS compared with the open group (10.7% versus 16%, respectively; *p* = 0.03). The benefit in terms of reduced incidence of complications was evident when specifically analyzing the incidence in the LS compared with the open group in terms of wound infection, postoperative ileus, biliary fistula, ascites, pleural effusion, lymphatic fistula, and arrhythmia.

The LS group, compared with the open group, showed a significant reduction in both median hospital stay and time for functional recovery (3 and 5 days, *p* = 0.032 and 4 and 6 days, *p* = 0.046 respectively). Furthermore, the interval time between surgery and subsequent adjuvant treatments (35 and 49 days median in the LS and the open group, respectively; *p* = 0.03) was significantly shorter in LS patients. A higher number of patients in the LS compared with the open group (82.7% and 77.3%) received systemic adjuvant therapies, without reaching statistical significance.

### 3.3. Long-Term Outcome Data

**Figure 2:**
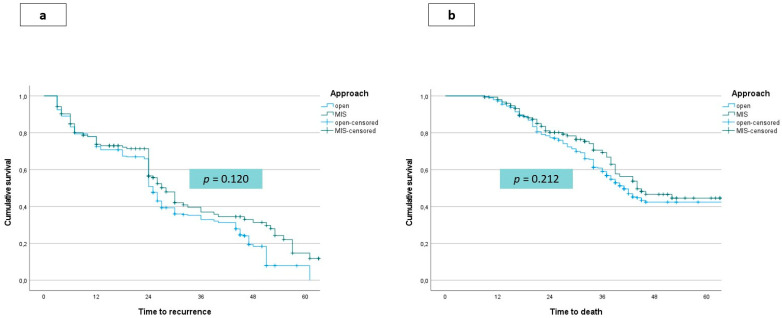
Survival functions. (**a**) Disease free survival according to treatment group (open versus laparoscopic). (**b**) Overall survival according to treatment group (open versus laparoscopic).

The median disease-free survival was 28 months (range 26–30) in the open and 32 months (range 28–35) in the LS group, with no significant difference. No differences were shown even in the comparison between the LS and the open group in terms of median overall survival (44 months, range 37–45 and 41 months, range 33–44, respectively). Kaplan–Meier curves for disease-free and overall survival according to the treatment group are shown in Figure 2a,b, respectively. Table 3 reports the incidence of recurrence and patterns of recurrence in the study and control groups. In particular, the disease recurrence rate was comparable in the LS compared with the open group (45.2% versus 56.7%), and the recurrence pattern was similar. Recurrence at the site of the resection margin, peritoneal seeding, or port-site metastases were not observed in any patients in the LS group. The treatment of recurrences was similar between the groups.

## 4. Discussion

The relative delay in the diffusion of the minimally invasive approach for iCCA has resulted in the current scarce availability of data on the oncological outcomes of this technique compared to the traditional open one [14] The present study is, to the best of our knowledge, the first to deal specifically with this issue: in two groups of patients homogeneously selected by propensity score matching, the LS technique proved to be similar in terms of the 3- and 5- year disease-free and overall survival to the open group. The most consistent advantages of LS are: first, the limitation of biological stress ensured by laparoscopy contributing to the maintenance of immune competence [20], while, conversely, immunosuppression linked to significant surgical stress greatly reduces the ability to respond to tumorigenesis and consequently increases the neoplastic risk, primarily that of relapse [21,22]. Secondly, the laparoscopic approach, making the postoperative course more favorable and reducing the time required for functional recovery, allows patients to start systemic chemotherapy programs more promptly [11,23]. In fact, although the overall number of patients undergoing adjuvant chemotherapy program is comparable between the two groups, in the laparoscopic group there is a shorter median surgery-to-start chemotherapy interval and a significantly higher number of patients who have started the cancer program within two and three months from surgical treatment.

The main challenge reported for the initial exclusion from minimally invasive programs of patients with iCCA is the need to perform a formal lymphadenectomy, which is required both for reasons related to the complete staging of the disease and for curative purposes [6,7,8,15,16]. Indeed, Bagante et al. reported a prognostic advantage from the removal of stations 8 and 12 during surgery [24]. The prerequisite for identifying an adequate lymphadenectomy is the removal of at least six lymph nodes [24,25]. In our previous experiments published in 2019, the effectiveness of laparoscopic compared to open lymphadenectomy in a mixed series of intrahepatic cholangiocarcinoma and gallbladder carcinoma was specifically analyzed. The number of lymph nodes collected was comparable in the laparoscopic and open series (8 versus 7, respectively; *p* = not significant); however, interestingly the percentage of patients who reached the AJCC recommended cut-off of six lymph nodes harvested was higher in the laparoscopic than in the open group. These results in the laparoscopic group were obtained without negatively affecting the overall postoperative morbidity and morbidity related to lymphadenectomy [7]. In a subsequent bi-institutional series including the results of a series of 208 patients, half of whom were treated by laparoscopic invasive approach and half by the open technique, the advantages of laparoscopy already described in other primary and secondary neoplasms of the liver were reconfirmed specifically in the iCCA, in a large series belonging to referral centers with HPB expertise [8]. In that series, the assessment of long-term outcomes was still affected by a relatively short observation period of the laparoscopic series, but a faster return to adjuvant programs had already been observed in the laparoscopic group [8]. In a recent meta-analysis by Regmi et al., eight studies were identified reporting the data of 552 patients operated on for laparoscopy and compared with 2320 open cases [26]. Considering data supporting the use of mini-invasiveness but still with significant limitations in terms of patients recruitment, the authors concluded that LS for iCCA is still in the early exploration phase, and consequently stronger evidence is needed to support its use and to draw conclusions, especially in terms of long-term outcomes [26].

The results of this series are important, since they not only confirm the non-inferiority of the laparoscopic technique in terms of long-term outcomes but also allow us to open a new scenario on the crucial value added by this technique in the natural history of patients with iCCA. In fact, patients with iCCA benefit from multimodal treatment, especially if negative prognostic factors such as multifocality and lymph node involvement are detected. Within a multidisciplinary discussion, the indication to start an adjuvant treatment is aimed to add a prognostic advantage in patients already treated with surgery. In the BILCAP study, a randomized, controlled, multicenter, phase 3 study conducted across 44 specialist hepatopancreatobiliary centers in the UK, patients were assigned to receive oral capecitabine versus observation [27]. In the per-protocol analysis, median recurrence-free survival was 25.9 months in the capecitabine group and 17.4 months in the observation group, strongly supporting the use of adjuvant therapies in resected patients. Interestingly, in this study patients who had not completely recovery from previous surgery were excluded and study eligibility to 16 weeks after surgery was allowed after two amendments (probably because too many patients needed to be excluded because of performance status in the interval of time closer to surgery) [27]. Consequently, there are no current data on the relationship between the timing of the initiation of chemotherapy and the impact on the risk of recurrence. However, it can be speculated that there is a correlation between early treatment initiation and oncological benefit. As already reported in other areas of oncological surgery of the liver, laparoscopy allows an earlier start of the adjuvant therapy also in cholangiocarcinoma [11,23]. Beyond the median surgery-adjuvant interval time, even the number of patients who had started the adjuvant treatment at two and three months after surgery were assessed; although the difference between the two approaches decreased over months, the LS group had a significantly higher number of cases with treatment initiation at both 2 and 3 months.

Furthermore, Sheka et al. reported that only 51% of patients who undergo up ront surgery actually receive NCCN-recommended adjuvant therapy [28]. It is therefore possible—and will be interesting to evaluate in the future—that the reduction in the biological impact of surgery allows a greater number of patients (who, due to the staging and characteristics of the disease, would have an indication for adjuvant therapies) to be able to start the scheduled program thanks to an adequate performance status. In fact, in Sheka et al. it was also reported how the receipt of surgery + chemotherapy was independently associated with survival on Cox proportional hazard ratio modeling compared to surgery alone or chemotherapy alone, and failure to administer multimodality therapy leads to sub-optimal outcomes for patients with node -positive biliary tract cancers [28].

This issue is remarkably relevant in patients with a high risk of VER. Tsimiligras et al. in fact reported that approximately one fourth of patients undergoing curative intent hepatectomy for iCCA develop VER, and that VER is associated with discouraging prognosis [29]. Obviously, in the presence of a very high preoperative risk of VER, the hypothesis of neoadjuvant chemotherapy must be considered by the multidisciplinary team. However, there might be a discrepancy between the preoperative and the postoperative risk score of VER, and this is not an infrequent situation. For instance, if a formal lymphadenectomy is performed allowing the identification of patients with positive lymph nodes even if they were not enlarged on CT scan or non-enhancing in PET, the postoperative score (i.e., the one used in this study) may indicate a high risk of VER, while the preoperative risk may be moderate. In these patients, the ability to start early treatment is crucial [29].

In conclusion, the laparoscopic approach for iCCA is once again confirmed to be associated with advantages in terms of intraoperative and short-term outcome, but also proves to be oncologically non-inferior to the open counterpart. In the present study, the overall and disease-free survival were found to be similar between the two. It is possible that laparoscopy can provide a specific benefit to patients with a higher risk of recurrence, for reasons that still need to be explained but that are likely related to the favorable biological scenario and to the possibility of starting the adjuvant chemotherapy program earlier.

## Figures and Tables

**Table 1:** Patients and disease characteristics after propensity score matching.

		LS Group (*n* = 150)	Open Group (*n* = 150)	*p*
Age (years)	*Mean ± SD*	61 ± 4	62 ± 7	ns
Male sex, *n* (%)		92 (61.3)	86 (57.3)	ns
ASA score, *n* (%)				ns
	1	33 (22)	31 (20.7)	
	2	79 (52.7)	83 (55.3)	
	3	38 (25.3)	36 (24)	
BMI	Mean ± SD	24.6 ± 2.6	24.9 ± 1.8	ns
Underlying liver disease, *n* (%)				ns
	None	105 (70)	114 (76)	
	Steatosis/mild impairment	25 (16.7)	23 (15.3)	
	Cirrhosis	20 (13.3)	13 (8.7)	
Previous abdominal surgery, *n* (%)				ns
	None	107 (71.3)	118 (78.7)	
	Yes, supramesocolic	32 (21.3)	20 (13.3)	
	Yes, inframesocolic	12 (8)	12 (8)	
CEA (ng/mL)	Mean ± SD	22 ± 21	35 ± 19	ns
Ca 19.9 (U/mL)	Mean ± SD	89 ± 76	93 ± 64	ns
Size (cm)	Mean ± SD	5.3 ± 2.3	5.8 ± 1.2	ns
Positive nodal status at imaging, *n* (%)		17 (11.3)	32 (21.3)	ns
Tumor number, *n* (%)				ns
	Single	108 (72)	105 (70)	
	Single with satellites	22 (14.7)	24 (16)	
	Multiple	20 (13.3)	21 (14)	
Histological grade, *n* (%)				ns
	Well	20 (13.3)	12 (8)	
	Moderate	107 (71.3)	122 (81.3)	
	Poor	25 (16.7)	16 (10.7)	
T stage, *n* (%) *				ns
	T1/T2	111 (74)	114 (76)	
	T3/T4	39 (26)	36 (24)	
Lymphadenectomy, *n* (%)		132 (88)	135 (90)	ns
Nodal status, *n* (%)				ns
	Negative	94 (62.7)	97 (64.7)	
	Positive	56 (37.3)	53 (35.3)	
Staging, *n* (%) *				ns
	I/II	91 (60.7)	93 (62)	
	III/IVa	59 (39.3)	57 (38)	
Preoperative CT, *n* (%)				ns
	Yes	5 (3.3)	7 (4.7)	
	No	145 (96.7)	143 (95.3)	
Suspected need for vascular resection, *n* (%)		2 (1.3)	3 (2)	ns
Suspected need for biliary resection, *n* (%)		5 (3.3)	6 (4)	ns
VER Low risk, *n* (%)		16 (10.7)	14 (9.3)	ns
VER Intermediate-risk, *n* (%)		85 (56.7)	83 (55.3)	ns
VER High risk, *n* (%)		49 (32.7)	53 (35.3)	ns

Abbreviations: ASA, American Society of Anesthesiology; BMI, Body Mass Index; CA, carbohydrate antigen; CEA, carcinoembryonic antigen; T, tumor; CT, chemotherapy; VER, very early recurrence; ns, not significant. * Defined according to the 8th Edition of the AJCC classification.

**Table 2:** Intra- and postoperative details. * Grade of complication was calculated according to the Dindo–Clavien classification of surgical complications [19].

		LS Group (*n* = 150)	Open Group (*n* = 150)	*p*
Procedure, *n* (%) *				ns
	Minor resection	99 (66)	95 (63.3)	
	Major resection	51 (34)	55 (36.7)	
Pringle Manuevre, *n* (%)				ns
	Not performed	17 (11.3)	14 (9.3)	
	Performed	133 (88.7)	136 (90.7)	
Lenght of surgery (min)	Mean ± SD	270 ± 65	230 ± 60	ns
Blood loss (mL)	Mean ± SD	150 ± 100	350 ± 250	0.021
Number of retrieved nodes	Median (range)	9 (5-11)	7 (5–14)	ns
Surgical margin, *n* (%)				ns
	R0	146 (97.3)	143 (95.3)	
	R1	4 (2.7)	7 (4.7)	
Conversion, *n* (%)		17 (11.3)	n.a.	
Surgical margin (mm)	Mean ± SD	6 ± 4	7 ± 4	ns
Intraoperative blood transfusions, *n* (%)				0.04
	No	144 (96)	138 (92)	
	Yes	6 (4)	12 (8)	
Postoperative blood transfusions, *n* (%)				0.03
	No	140 (93.3)	128 (85.3)	
	Yes	10 (6.7)	22 (14.7)	
Complications, *n* (%)				
	Hemorrhage	0	3 (2)	ns
	Wound infection	2 (1.3)	6 (4)	0.02
	Ileus	2 (1.3)	7 (4.7)	0.01
	Biliary fistula	6 (4)	12 (8)	0.03
	Transient liver failure	4 (2.7)	7 (4.7)	ns
	Ascites	10 (6.7)	16 (10.7)	0.03
	Pleural effusion	10 (6.7)	14 (9.3)	0.04
	Pneumonia	2 (1.3)	4 (2.7)	ns
	Fever	6 (4)	9 (6)	ns
	Pancreatitis	3 (2)	3 (2)	ns
	Lymphatic fistula	3 (2)	10 (6.7)	0.03
	Arrhythmia	4 (2.7)	6 (4)	0.04
	DVT/PE	2 (1.3)	3 (2)	ns
Overall complication rate, *n* (%)		22 (14.7)	36 (24)	0.015
Grade of complications, *n* (%) *				0.03
Minor	I grade	4 (2.7)	7 (4.7)	
	II grade	12 (8)	17 (11.3)	
Major	III grade	4 (2.7)	9 (6)	ns
	IV grade	2 (1.3)	3 (2)	
Mortality, *n* (%)		2 (1.3)	2 (1.3)	ns
Functional recovery (days)	Median (range)	3 (1–5)	5 (3–10)	0.032
Lenght of stay (days)	Median (range)	4 (2–10)	6 (3–21)	0.046
Interval surgery-adjuvant treatment (days)	Median (range)	35 (30–55)	49 (37–63)	0.03
Systemic adjuvant therapy, *n* (%)		124 (82.7)	116 (77.3)	0.05

Abbreviations: DVT, deep vein thrombosis; PE, pulmonary embolism; ns, not significant.

**Table 3:** Long-term outcomes.

		LS Group (*n* = 150)	Open Group (*n* = 150)	*p*
Disease recurrence (*n*) %		89 (59.3)	95 (63.3)	ns
Modality of recurrence, *n* (%) *				ns
	Nodal	17 (19.1)	19 (20)	
	Intrahepatic, monofocal	19 (21.3)	18 (18.9)	
	Intrahepatic, multifocal	47 (52.8)	51 (53.7)	
	Extrahepatic	26 (29.2)	31 (32.6)	
Therapy of recurrence, *n* (%) *				ns
	Re-resection	11 (12.4)	10 (10.5)	
	Medical therapy	69 (77.5)	76 (80)	
	Other local treatments	13 (14.6)	15 (15.8)	

* Percentage calculated on the number of patients who developed a recurrence of the disease.

## Data Availability

The data presented in this study are available on request from the corresponding author. The data are not publicly available due to privacy reasons.
